# Supplementary material for: Multi‐dataset identification of innovative feature genes and molecular mechanisms in keratoconus
Source: J Cell Mol Med. 2024 Sep 19;28(18):e70079. doi: 10.1111/jcmm.70079 (PMC11412914; doi:10.1111/jcmm.70079)
Supplement: Supplementary file 2 — Material S2. [file JCMM-28-e70079-s002.zip › 7.GSEA analysis/5.RNF217-AS1/KEGG_REGULATION_OF_AUTOPHAGY.html]

Details for gene set KEGG\_REGULATION\_OF\_AUTOPHAGY[GSEA]

|  || Dataset | RNF217-AS1.group.cls#h\_versus\_l |
| Phenotype | group.cls#h\_versus\_l |
| Upregulated in class | h |
| GeneSet | KEGG\_REGULATION\_OF\_AUTOPHAGY |
| Enrichment Score (ES) | 0.3266771 |
| Normalized Enrichment Score (NES) | 1.0102376 |
| Nominal p-value | 0.42540324 |
| FDR q-value | 1.0 |
| FWER p-Value | 0.999 |
Table: GSEA Results Summary

  

Fig 1: Enrichment plot: KEGG\_REGULATION\_OF\_AUTOPHAGY      
 Profile of the Running ES Score & Positions of GeneSet Members on the Rank Ordered List

  

| SYMBOL | TITLE | RANK IN GENE LIST | RANK METRIC SCORE | RUNNING ES | CORE ENRICHMENT || 1 | IFNA14 | na | 927 | 0.451 | 0.0937 | Yes |
| 2 | IFNA4 | na | 1517 | 0.391 | 0.1812 | Yes |
| 3 | IFNA16 | na | 1944 | 0.354 | 0.2634 | Yes |
| 4 | IFNA6 | na | 2619 | 0.310 | 0.3267 | Yes |
| 5 | IFNA7 | na | 9097 | 0.103 | 0.1676 | No |
| 6 | IFNA21 | na | 11545 | 0.062 | 0.1136 | No |
| 7 | IFNA2 | na | 12465 | 0.049 | 0.1002 | No |
| 8 | ATG4D | na | 12547 | 0.048 | 0.1107 | No |
| 9 | GABARAPL1 | na | 12551 | 0.048 | 0.1234 | No |
| 10 | ATG4B | na | 13852 | 0.032 | 0.0946 | No |
| 11 | ULK1 | na | 15064 | 0.018 | 0.0645 | No |
| 12 | BECN1 | na | 16090 | 0.008 | 0.0371 | No |
| 13 | ATG7 | na | 17880 | -0.010 | -0.0119 | No |
| 14 | ULK2 | na | 18031 | -0.011 | -0.0132 | No |
| 15 | GABARAPL2 | na | 20398 | -0.034 | -0.0724 | No |
| 16 | INS | na | 20462 | -0.035 | -0.0650 | No |
| 17 | GABARAP | na | 20584 | -0.036 | -0.0589 | No |
| 18 | ATG5 | na | 21857 | -0.049 | -0.0824 | No |
| 19 | PIK3C3 | na | 22999 | -0.062 | -0.0988 | No |
| 20 | ATG4A | na | 23361 | -0.066 | -0.0914 | No |
| 21 | ATG3 | na | 23506 | -0.068 | -0.0773 | No |
| 22 | ULK3 | na | 23542 | -0.069 | -0.0599 | No |
| 23 | PRKAA1 | na | 23950 | -0.074 | -0.0518 | No |
| 24 | ATG12 | na | 24614 | -0.084 | -0.0484 | No |
| 25 | ATG4C | na | 24780 | -0.087 | -0.0299 | No |
| 26 | PIK3R4 | na | 25416 | -0.097 | -0.0222 | No |
| 27 | PRKAA2 | na | 29109 | -0.179 | -0.0808 | No |
| 28 | IFNA8 | na | 29826 | -0.201 | -0.0477 | No |
| 29 | IFNA10 | na | 32398 | -0.317 | -0.0373 | No |
| 30 | IFNG | na | 33359 | -0.391 | 0.0395 | No |
Table: GSEA details [plain text format]

  

Fig 2: KEGG\_REGULATION\_OF\_AUTOPHAGY      
 Blue-Pink O' Gram in the Space of the Analyzed GeneSet

  

Fig 3: KEGG\_REGULATION\_OF\_AUTOPHAGY: Random ES distribution      
 Gene set null distribution of ES for **KEGG\_REGULATION\_OF\_AUTOPHAGY**
